# Blood Biomarkers from Research Use to Clinical Practice: What Must Be Done? A Report from the EU/US CTAD Task Force

**DOI:** 10.14283/jpad.2022.85

**Published:** 2022

**Authors:** D. Angioni, J. Delrieu, O. Hansson, H. Fillit, P. Aisen, J. Cummings, J.R. Sims, J.B. Braunstein, M. Sabbagh, T. Bittner, M. Pontecorvo, S. Bozeat, J.L. Dage, E. Largent, S. Mattke, O. Correa, L.M. Gutierrez Robledo, V. Baldivieso, D.R. Willis, A. Atri, R.J. Bateman, P-J. Ousset, B. Vellas, M. Weiner, EU/US/CTAD Task Force

**Affiliations:** 1. Gerontopole of Toulouse, Alzheimer’s Disease Research and Clinical Center, Toulouse University Hospital, Toulouse, France; 2. Memory Clinic, Skåne University Hospital, Malmö, Sweden; 3. Alzheimer’s Drug Discovery Foundation, New York, NY, USA; 4. USC Alzheimer’s Therapeutic Research Institute, San Diego, CA, USA; 5. Chambers-Grundy Center for Transformative Neuroscience, Department of Brain Health, School of Integrated Health Science, University of Nevada Las Vegas (UNV), Las Vegas, Nevada, USA; 6. Lilly, Indianapolis, USA; 7. C(2)N Diagnostics, Saint Louis, MO, USA; 8. Barrow Neurological Institute, Phoenix Arizona, AZ, USA; 9. Genentech, A Member of the Roche Group, Basel, Switzerland; 10. Eli Lilly and Company, Indianapolis, IN, USA; 11. Hoffmann-La Roche, Basel, Switzerland; 12. Indiana University School of Medicine, Departement of Neurology, Indianapolis, IN, USA; 13. Department of Medical Ethics and Health Policy, University of Pennsylvania Perelman School of Medicine, Philadelphia, USA; 14. Center for Economic & Social Research, University of Southern California, Los Angeles, USA; 15. GRIS - Gestão Responsável Integrada de Saúde, Rio de Janeiro, Brazil; 16. National Institute of Geriatrics, Mexico City, Mexico; 17. AdventHealth Memory Care Clinic, Winter Park, USA; 18. Department of Family Medicine, Indiana University School of Medicine, Indianapolis, USA; 19. Banner Sun Health Research Institute, Banner Health, Sun City, USA; 20. Department of Neurology, Brigham and Women’s Hospital and Harvard Medical School, Boston, Massachusetts, USA; 21. Washington University School of Medicine, St. Louis, MO, USA; 22. UNM Division of General Internal Medicine and Geriatric Medicine, Albuquerque NM, USA; 23. University of California, San Francisco, CA, USA

**Keywords:** Alzheimer’s disease, blood biomarkers, diagnostic, clinical trials, amyloid, p-tau, neurofilament light

## Abstract

Timely and accurate diagnosis of Alzheimer’s disease (AD) in clinical practice remains challenging. PET and CSF biomarkers are the most widely used biomarkers to aid diagnosis in clinical research but present limitations for clinical practice (i.e., cost, accessibility). Emerging blood-based markers have the potential to be accurate, cost-effective, and easily accessible for widespread clinical use, and could facilitate timely diagnosis. The EU/US CTAD Task Force met in May 2022 in a virtual meeting to discuss pathways to implementation of blood-based markers in clinical practice. Specifically, the CTAD Task Force assessed: the state-of-art for blood-based markers, the current use of blood-based markers in clinical trials, the potential use of blood-based markers in clinical practice, the current challenges with blood-based markers, and the next steps needed for broader adoption in clinical practice.

## Introduction

Timely and accurate diagnosis of Alzheimer’s disease (AD) in clinical practice is a great challenge. Currently, the clinical diagnosis of AD is poor; about 20-25% of the patients are misdiagnosed if cerebrospinal fluid (CSF) or positron emission tomography (PET) biomarkers are not used (1, 2). This misdiagnosis is particularly important during early disease stages with subtle or mild symptoms (subjective cognitive decline [SCD] and mild cognitive impairment [MCI]) and in primary care, where more than 50% of patients with cognitive impairment are not recognized or correctly diagnosed (2). This misdiagnosis results in suboptimal treatment and care, delayed or incorrect therapies, and inaccurate information about disease and prognosis (3).

In 2021, on the basis of the dramatic effect on cerebral amyloid-plaque load, aducanumab was granted accelerated approval for the treatment of AD in the United States (US) and phase III trials with other anti-amyloid monoclonal antibodies are ongoing (gantenerumab, donanemab, lecanemab) (4). If these potentially disease-modifying anti-amyloid therapies become approved and widely available for use in clinical practice, it will become even more important to establish an accurate early diagnosis. To date, PET and CSF biomarkers are the most widely used biomarkers in clinical practice but easily accessible, cost-effective, and accurate biomarkers such as blood-based markers (BBM) have the potential for widespread clinical use, including in primary care (3).

However, several issues remain to be overcome before BBM can be expected to represent a routine component of clinical care. A consensus on progress, barriers and next steps is needed to make efficient advances in the field.

The EU/US CTAD Task Force met in May 2022 in a virtual meeting to discuss the pathways to implementation of BBM use in clinical practice. In this objective, the CTAD Task Force assessed:

1. The state-of-scientific development of BBM.
2. The current use of BBM in clinical trials.
3. Limitations of the currently available data and gaps to be resolved
4. The potential use of BBM in clinical practice.
5. The next steps for broader adoption of BBM in clinical practice.

## State-of-scientific development of blood-based markers

### Amyloid and p-tau blood-based markers in AD

Plasma Aβ42/40 ratio, p-tau, serum neurofilament light chain (NfL) and glial fibrillary acidic protein (GFAP) are the most advanced BBM developed for the diagnosis and prognosis of AD and to potentially monitor the effects of disease-modifying therapies (DMTs).

There are many assays to measure Aβ in blood and to detect positive amyloid status in patients with early AD. Plasma Aβ42/Aβ40 ratio, measured by high-precision assay, can detect abnormal amyloid status in cognitive impaired (5, 6), and cognitively unimpaired subjects even before amyloid PET reach its threshold of positivity (7). Due to their high-precision and robust implementation, mass spectrometry-based (MS) methods and fully automated immunoassay methods are considered to have the best opportunity for clinical implementation in routine care; MS methods have been implemented as lab developed tests (LDTs) in the United States (8).

Similarly to Aβ isoforms, several forms of p-tau such as p-tau181, p-tau217 and p-tau231 can be detected in plasma with high accuracy (9–11). Plasma p-tau217 correlates with tau tangles in AD, but not in non-AD pathologies (12). P-tau217 levels appear to be elevated in the presence of amyloid plaques but not in the absence of amyloid plaques as occurs in primary age related tauopathy (PART) or other non-AD tauopathies (e.g., progressive supranuclear palsy or corticobasal degeneration) (13). Plasma p-tau217 levels are increased by 300-700% in symptomatic AD and can differentiate AD from non-AD diseases with an accuracy similar to CSF p-tau and Tau PET (12). In cognitively unimpaired subjects, plasma p-tau217 with or without Aβ42/40 ratio can predict cerebral amyloid pathology assessed by amyloid PET (14). Plasma p-tau217 can also detect AD pathology in cognitively unimpaired subjects. Its level is increased by 80-350% in cognitively unimpaired subjects with positive amyloid status and changes up to 20 years before onset of cognitive decline (12, 15). In non-demented subjects with cognitive complaints, individual probability of conversion to AD has been assessed by a model combining plasma p-tau, apolipoprotein E (APOE) ε4 genotype, and simple executive (animal fluency and trail making test part B) and memory (Alzheimer’s Disease Assessment Scale–Cognitive Subscale memory subtests) tests. This model predicted dementia better than a clinical assessment performed by specialists in memory disorders and performed similarly to CSF biomarker-based algorithms (16).

### Robustness of blood-based markers in clinical routine

There is a theoretical concern that plasma Aβ42/40 ratio may have a lower diagnostic accuracy than the Aβ42/40 ratio in CSF, representing a potential problem for clinical robustness. Indeed, Aβ42/40 ratio is reduced by only 8-15% in blood while Aβ42/p-tau ratio is reduced by more than 40-60% in CSF, making it challenging to apply a cutoff at the individual level. From the Alzheimer’s Disease Neuroimaging Initiative (ADNI, n = 118 from cognitive unimpaired to dementia subjects) cohort, the performance of BBM has been evaluated by a theoretical simulation evaluating an increase in the inter-assay coefficient of variability (17). The robustness of plasma Aβ42/40 ratio is quickly reduced with an increase of inter-assay variability. An increase of only 5% in coefficient of variability impacted its accuracy for discriminating amyloid positive PET versus negative cases. Cullen et al. measured the test-retest variability (random effect) of blood Aβ42/Aβ40 ratio, NfL, GFAP, and p-tau217 from the Biomarkers For Identifying Neurodegenerative Disorders Early and Reliably (BioFINDER) -1 study (n=399) (18). In this study, the test-retest variability reduced the performance of biomarkers for predicting abnormal amyloid accumulation and progression to AD dementia. Compared to the other analytes, p-tau217 was less affected by test-retest variability. Direct head-to-head studies have demonstrated that high precision and accurate plasma Aβ42/Aβ40 ratios perform as well or better than immunoassay measures (19) in identifying AD pathology. For example, a study of five different assays compared in the same cohort, the IP-MS plasma Aβ42/Aβ40 assay outperformed all immunoassays compared to amyloid PET or CSF Aβ as the reference standard (8). Multiple international studies, some with longitudinal collection and conducted over several years, have consistently shown performance characteristics of receiver operator curve (ROC) area under the curve (AUC) of ~ 0.85 (5–7, 20–22). These performances are similar to several blood plasma p-tau immunoassays, and with rigorous stability that provides a benefit to aid in the diagnosis of AD. Further, results from Cullen et al. (18) shown that combining BBM reduced the impact of assay variability on biomarker performance. Recently, it was shown that in cognitive impairment at the earliest stages of pathological amyloid accumulation, the combination of p-tau217 with Aβ42/40 may be more sensitive than p-tau217 alone for the detection of AD (23). Combined BBM could be used in clinical practice and can assist in avoiding misdiagnoses.

### Confounding factors

Performances of p-taul81 and p-tau217 have been studied in heterogeneous community-based populations from the Mayo Clinic Study of Aging (n = 1329). This analysis showed the effect of comorbidities on plasma p-tau181 and p-tau217 levels. Increase in p-tau181 and p-tau217 were associated with age especially in subjects with a positive amyloid status. Several comorbidities including chronic kidney disease, hypertension, stroke, and myocardial infarction were associated with higher plasma p-tau181 and p-tau217 levels, even in an age- and sex-adjusted model (24). In BioFINDER-1 and -2 studies, plasma NfL, GFAP and p-tau levels were affected by kidney function and body mass index, but these potential confounding factors seem to have only small effects on the clinical performance of the biomarkers. Based on these data, a significant research priority is to better understand the effect of comorbidities on BBM for their proper interpretation in clinical practice.

A secondary analysis from the ADNI study shown that sex may impact the clinical interpretation of plasma p-tau181 concentrations (25). In women, high plasma p-tau levels were associated with worse phenotypic markers (i.e., greater cortical Aβ deposition, higher CSF p-tau181 levels, lower brain glucose metabolism) compared with men. Moreover, higher baseline p-tau levels in women were associated with greater risk of progression to dementia and faster cognitive decline, compared with men.

Race and ethnicity are also pertinent demographic variables that may impact the interpretation of BBM data in the clinical setting. A recent evaluation studied the performance of various BBM in a cohort of 76 matched pairs (91% cognitively normal) of African-American (AA) and Non-Hispanic White individuals (NHW) (26). The results suggested that a high-performance plasma Aβ42/40 assay may provide a consistent and reliable measure of brain amyloidosis across AA and NHW groups, but models based on plasma p-tau181, p-tau231, and NfL, may perform inconsistently and could result in a disproportionate misdiagnosis of AA individuals. Special attention should be given to the use of algorithms including BBM and APOE genotype in populations without a predominant European ancestry. Indeed the association between APOE alleles and AD risk varies across different ethnic groups (27, 28). Additional studies are necessary to understand how to best interpret BBM results across racially and ethnically diverse populations for the benefit of clinical care of these patients.

### Limitations

Despite the encouraging data available thus far, it should be emphasized that most of the data reviewed above come from previously collected, retrospective studies, where the plasma samples were analyzed in large batches, applying optimal cut points derived from batch data, and evaluating AUCs derived from these cut points. There are few prospective data available where plasma samples were obtained longitudinally over a long period of time and clinical performances calculated from a predetermined cut point. Such data are being developed through clinical studies and are recommended before implementation of any particular BBM or combination of BBM in clinical practice.

## Current use of plasma biomarkers in clinical trials

The introduction of new BBM for AD will improve the design and conduct of clinical trials evaluating DMTs. For the screening of these trials, BBM can help identify individuals with a high probability of not having AD pathology. This approach is of particular interest in preclinical trials in AD and could significantly reduce the number of negative CSF and PET scans commonly seen in the screening phases of trials for putative DMTs (29).

Within the framework of clinical trials, three potential uses of BBM can be identified:

1. As pre-screening tools to eliminate individuals who are likely to be amyloid negative with CSF or PET to reduce the number of required confirmatory CSF and PET testing
2. As an inclusion criterion (diagnosis tool) without confirmatory CSF or PET testing
3. As pharmacodynamic markers to detect direct target engagement and monitor disease-modifying effects

### Blood-based markers as screening tools

This use of BBM as a pre-screening tool is currently the most common. The obvious goal is to increase the prevalence of amyloid positive individuals before they undergo CSF or PET testing, which are needed to confirm the diagnosis of AD for final inclusion decisions for trials. This approach makes it possible to avoid unnecessary examinations and therefore reduce the cost and burden associated with these invasive and costly examinations. Biomarkers currently used in this pre-screening perspective include the Aß42/Aß40 ratio (30), p-tau181, p-tau217 (31), or algorithms considering, in addition to the biomarker, data such as age and APOE status (32, 33). Currently available data support the utility of plasma prescreening: for example, the preliminary analysis of the AHEAD 3-45 study demonstrated an improvement of positive predictive value from 28.9% PET eligible to 61.5% PET confirmed with a plasma prescreen (30). However, the value of BBM as screening tools prospectively needs to be confirmed.

### Blood-based markers as an inclusion criterion

Use as the main inclusion criterion for trials, indicating Alzheimer’s pathology, is conceivable only if the BBM can achieve sufficiently high diagnostic performance. This is particularly advantageous for trials where CSF or PET is not required to demonstrate drug target engagement or pharmacodynamic response in each participant (34). Currently, only one trial, TRAILBLAZER-ALZ3, evaluating the efficacy of donanemab in cognitively normal people with elevated plasma p-tau 217, uses this recruitment strategy. This approach is based on the good discriminating performance established by the p-tau217 assay (12). This strategy seems particularly attractive for this type of trial including participants at the preclinical stage of AD, where participants are less likely to accept invasive diagnostic tests. However, at the current time there are limited data concerning the value of BBM used prospectively as inclusion criteria.

### Blood-based markers as pharmacodynamic markers

BBM could also be used as pharmacodynamic markers to detect direct target engagement and disease modifying effects. Both effects are critical for identifying an appropriate dose and increasing confidence that the treatment leads to disease-relevant outcomes.

BBM could have utility as surrogate endpoints, resulting in shorter clinical trials or smaller sample sizes. In the early stages of drug development, they could help make go/no go decisions for later larger trials of drug candidates, before effects are demonstrated on measures of cognition or function (34). Plasma Aß42/Aß40 ratio has reflected amyloid removal, corroborated with amyloid PET, in several drug trials of amyloid-removing antibodies including gantenerumab and lecanemab (35, 36). There are promising data from epidemiological studies showing that plasma p-tau has properties that may make it suitable as a pharmacodynamic marker, including longitudinal increases in preclinical and prodromal stages, and longitudinal correlations with brain structure and cognitive measures (37). In the treatment arms of EMERGE and ENGAGE trials of aducanumab (4) a reduction of p-tau181 levels was observed over time in concert with amyloid plaque lowering. Conversely, p-tau181 levels increased in the placebo groups. A greater reduction in plasma p-tau181 levels was associated with less clinical decline. Moreover, reduction in plasma p-tau181 levels was correlated with reductions in amyloid PET standard uptake value ratio (SUVR). Additionally, in exploratory analysis of TRAILBLAZER-ALZ trial, donanemab showed a reduction of plasma p-tau217 levels over time. P-tau217 reduction was correlated with changes in amyloid and tau load as assessed by PET (press release July 2021). Recently, the Alzheimer’s Association recommended use of BBM for the prescreening or screening of AD subjects in DMT trials before confirmation by PET and CSF testing (38). Table 1 summarizes the current use of plasma biomarkers in ongoing disease-modifying trials.

## Blood biomarkers in clinical practice

### From lab to clinical practice

Advancing BBM to clinical application may improve disease detection and patient care in the coming years. The scalability of a biomarker, from research to clinical practice, requires the application of 3R’s in its development: AccuRacy, Robustness and Regulation. AccuRacy: clinical performance is demonstrated in the population in which the test will be used in clinical practice. Robustness: ability to apply a predefined cut-off prospectively while maintaining clinical performance despite differences in sample handling, operators, and laboratories. Regulation: demonstration of the analytical and clinical performance of a test before it is brought to the market. As discussed above, the ability of BBM to be used in clinical practice will critically depend on the results of prospective trials demonstrating assay stability and the robustness of predetermined cut points.

### Which marker in clinical practice?

Plasma p-tau markers vary in their capability to predict AD according to the specific protein species considered (i.e., p-tau217, p-tau181 and p-tau231), the assay, and thresholds used (10, 12, 24). A recent comparison study showed a relatively high correlation between three p-tau181 assays (Eli Lilly, ADx, Quanterix), one p-tau217 assay (Eli Lilly), and one p-tau231 assay (Gothenburg) (10). Recently it was shown that the p-tau217 assay developed by Janssen exhibits similar performance to the one developed by Eli Lilly (39).

The PrecivityAD^™^ test is a tandem MS assay used to quantify Aβ42 and Aβ40 isoforms and determine APOE genotype (32). The PrecivityAD test is intended for use in subjects older than 60 years with MCI or dementia. The PrecivityAD test output is the Amyloid Probability Score (APS), an algorithm derived from the Aβ42/40 ratio, the APOE genotype and the patient’s age. The APS reflects the likelihood that a patient, on a scale of 0-100, will be amyloid positive on an amyloid PET scan. In two studies, the PARIS study (ClinicalTrials.gov Identifier: NCT02420756) and the Mission AD study (ClinicalTrials.gov Identifier: NCT02956486), involving 686 participants, the APS showed high concordance with amyloid PET status with an area under the curve of 0.88 and an overall accuracy of 81% (6). The cutoff values were established to balance the tradeoff between low frequency of intermediate results and high accuracy (6). Three APS categories were defined: low APS score (0-35) consistent with a low likelihood of amyloid plaques; intermediate APS score (36-57) unable to distinguish between the presence or absence of amyloid plaques; high APS score (58-100), consistent with a high likelihood of amyloid plaques. Importantly, 86% of patients fell into the actionable low or high APS score categories. The PrecivityAD assay is now being used prospectively with predetermined cut points. Interim results from a study underway (NCT05477056) are encouraging in supporting an association of the APS with improved AD diagnostic confidence and clinical decision making. However, at the current time, there are few published data available demonstrating the robustness of the value of the PrecivityAD test in a prospective setting.

The Quest AD-Detect^™^ plasma biomarker test provides an Aβ42/40 ratio and uses a cut off score of 0.160. Ratios ≥0.160 suggest a lower-than-normal risk of AD and the need to investigate for non-AD causes of cognitive impairment in symptomatic individuals. Ratios <0.160 are consistent with AD and indicate the need for further evaluation to verify the AD diagnosis. This assay and the threshold score require prospective validation.

NfL is a neuron-specific filament protein that has recently emerged as a biomarker of neuronal injury. The neurofilament proteins levels increase in CSF and in blood, following neuroaxonal damage and regardless of causal pathways. NfL levels increase with aging (40). They are associated with cognitive status and can potentially predict cognitive decline in multiple neurodegenerative conditions such as cerebral small-vessel disease, dementia with Lewy bodies, dementia due to Parkinson disease, frontotemporal dementia, and multiple sclerosis (41). Although Immunoblot and enzyme-linked immunosorbent assay provided limited sensitivity, third (electrochemiluminescence) and fourth-generation (single-molecule array) assays permit highly sensitive detection in blood samples (41). Recently, the Elecsys Amyloid Plasma Panel (EAPP), developed by Roche, which measures p-tau181 and APOE4 in an individual’s blood sample earned breakthrough label from Food and Drug Administration. This is an important step toward more widespread availability of these biomarkers.

### What objective?

The different roles of BBM are summarized in Table 2. In clinical practice, BBM may assist in diagnosis and prognosis. In the future, in individuals diagnosed with AD and treated with new DMTs, BBM would be useful to monitor the changes related to the treatment. In this context, BBM might also be essential for the safe and effective use of the treatment. Moreover, BBM would help to identify patients who are most likely to benefit from the treatment as well as individuals at increased risk of serious adverse reactions because of treatment. The same biomarkers can play different roles, especially if different thresholds are used (e.g., lower thresholds to screen patients for additional evaluation as PET or CSF, and higher thresholds to confirm the presence of the disease).

### Where?

If it is assumed that BBM are demonstrated to be stable and robust in a prospective setting, BBM could be used in primary care. A possibility is to utilize BBM in primary care to identify the patients with cognitive impairment that need to be referred for further cognitive evaluation by specialists in memory disorders. For this reason, a biomarker of neurodegeneration or one not specific to AD, such as the NfL, may represent a good candidate to be implemented in primary care. However, the robustness of this strategy in primary care needs to be determined. Another possibility is to recommend that trained primary care physicians can use AD specific BBM, including Aß42/Aß40 ratio and p-tau measures, for diagnosis and management without referral to a memory disorder specialist. The following arguments support this option:

- Timely detection of cognitive impairment, and accurate etiologic diagnosis are notoriously poor in primary care clinics due to several challenges including the lack of simple, accurate and accessible tests and the scarcity of clinicians trained in cognitive disorders. BBM are reaching performance levels similar to PET or CSF and are now available with improved versions coming soon.
- In many areas, most AD care is still provided by non-memory specialists, even more so in regions with low access to specialty care.
- If AD-specific treatments (e.g., anti-amyloid) are approved and become widely accessible, the need for detection and accurate diagnosis will be amplified.

In the last decade, digital technologies (e.g., data collection application, sensors, wearable devices) that allow remote assessment and collection of real-world data on cognitive outcomes have attracted great attention and interest in the field of cognitive diseases (42, 43). The World Health Organization (WHO) ICOPE step 1 is a simple tool, which can be used to detect an impairment in one or more domain(s) of the intrinsic capacity (i.e., cognition, mood, nutrition, mobility, sensory). In France, a mobile app and a conversational robot, that enable self-assessment, have been developed to promote ICOPE step 1 use (44). These tools are now extended internationally. In ICOPE step 1, memory is assessed by a subjective question, an orientation task, and a free recall of 3 words. In a population of about 18000 individuals with a mean age of 76 years old, it was shown that 60% presented either a subjective or an objective complaint (45). In 50 % of cases these alterations were persistent after 6 months (not published data). In primary care, digital biomarkers can be used to preselect individuals at risk of cognitive decline that could benefit from BBM assessment. In Mexico, the ICOPE step 1 is followed by the Davos Alzheimer’s Collaborative program to explore the addition of a BBM in a real-world setting, but under a research protocol, to assess facilitators and barriers to such an approach from a health system viewpoint.

In the specialty care setting, the goal of the BBM is the etiologic diagnosis of dementia and MCI. For this purpose, Aß42/40 ratio, p-tau217 and multiplex assays would be performed as part of (not replacement for) a amyloid, tau, neurodegeneration (AT(N)) classification system (46). A great benefit of a BBM in specialty care would be to reduce unnecessary confirmatory procedures as PET and CSF biomarkers. In this way, the specialty care pathway would be more streamlined and made more efficient.

### When?

In the first step, BBM would be collected only in subjects with an objective cognitive impairment (MCI and dementia). BBM should be ordered as part of the initial diagnostic workup, unless other (non-AD) obvious and untreated causes of cognitive decline are present. Since the first signs of cognitive decline and in case of suspicion of AD, BBM can be conducted in parallel with history, collection of risk factors and medications, cognitive and behavioral status exam, medical/neurologic exam and brain structural imaging. BBM will also have great utility in patients who have contraindications to lumbar puncture or in those reluctant to lumbar puncture.

### How?

For diagnosis, BBM could be useful when high confidence is required for AD or non-AD pathologies. For the diagnosis of typical amnesic AD, BBM, especially p-tau217, could be sufficient or could be part of a multiplex assessment with Aß42/40 and APOE genotype. For atypical presentations (non-amnesic, early onset, rapidly progressive, logopenic aphasia, posterior cortical atrophy), BBM for AD are likely sufficient if positive; but if negative, and AD remains in the differential diagnosis with at least moderate likelihood, suggest the need for CSF biomarkers or FDG-PET. In case of discordance of amyloid and tau BBM or intermediate results, CSF may resolve the uncertainty (or PET if CSF is contraindicated, Table 3). The results of BBM should be integrated with all other clinical features in a patient-centric manner. Even in the case of AD blood biomarkers positivity, clinician should not overlook other potentially contributing factors and conditions (e.g., depression, obstructive sleep apnea, iatrogenic etiologies).

### Advantages of blood-based markers in health care systems

Previous work has suggested that health systems are ill-prepared to cope with the expected workload of new treatments: the prevalent caseload is large and the number of dementia specialists is not sufficient. Thus, the wait time for comprehensive cognitive evaluations is likely to increase (47). One strategy to facilitate the diagnosis of AD and potentially to facilitate access to new treatments would therefore be to optimize triage in primary care. A recent study estimated impact of BBM on wait times and cost with a simulation model in several scenarios (Mini Mental State Examination MMSE only, BBM only, combination of both) (48). The combination of MMSE and BBM could strongly decrease the demand for specialist visits, the average wait time to complete the diagnosis process and the average annual cost by $400 to $700 million for the U.S. health care system. Carefully and appropriately implementing of a similar strategy, combining MMSE (or a similar brief validated cognitive test e.g., MoCA) and BBM in primary care settings, could also potentially improve the diagnosis accuracy; while utilizing a BBM only strategy would lead to many inappropriate referrals of cognitively unimpaired individuals to specialists in memory disorders.

The BBM have the potential for being more accessible and cheaper (less invasive, time-intense, costly, infrastructure dependent, and resource requiring – can be done in the community setting) than CSF and PET biomarkers. They could improve detection, diagnostic accuracy, patient-centered autonomy and empowerment, and better overall care. The use of BBM in clinical practice would be a great opportunity for referral to research (e.g., use of legacy samples and screening for clinical trials). Programs, such as the Davos Alzheimer’s Collaborative are exploring this workflow in a real-world setting, but under a research protocol, to assess facilitators and barriers to such an approach from a health system viewpoint.

### Potential risks of blood-based markers use in clinical practice

Among the risks, discussed above, there are lack of stability of BBM assay results when used prospectively, lack of robustness of cut points determining normal/abnormal results and the limited assessment of these cut points/ranges in real-world populations with ethnocultural diversity, advanced age (oldest old), and comorbidities. Attention to strict quality control measures over time is essential to mitigate part of this risk. These issues must also be resolved prior to implementation in clinical practice. In the absence of consensus guidelines regulating the use of BBM, there is an important risk of misuse and misinterpretation of the results. A common example of misuse is the use of BBM in cognitive unimpaired individuals, especially without associated cognitive testing, when the BBM has not been validated for this application. An important risk is the underdiagnosis of non-AD pathologies and other conditions potentially contributing to cognitive disorder in patients with negative BBM. Indeed, unexperienced users could interpret negative values as supporting a lack of need for further assessment (for other causes and contributors) and to refer patients to specialists in memory disorders. On the contrary, positive values could lead unexperienced BBM users to make a diagnosis of AD while disregarding other likely causes (e.g., co-existing vascular cognitive impairment or Lewy Body Disease) and potentially contributing conditions.

### Ethical considerations

Being minimally invasive, cost-effective, and globally scalable, BBM represent an opportunity to improve equitable access to medical care and research and to limit social disparities in healthcare. Patients throughout the United States and other regions of the world do not have easy and uniform access to specialized care centers, which are often the locations for PET imaging or specialists that can perform lumbar punctures for CSF analysis. These strategies are largely concentrated in major metropolitan areas, which present substantial geographic access barriers for many patients. Research has demonstrated that PET scan usage is associated with challenges in reaching healthcare equity and inclusion (49). Studies indicate that learning biomarker results is safe, and that patients understand their results correctly (50, 51). However, these data came from motivated populations of research participants (52, 53) and the disclosure of results was conducted by experienced providers; thus, they may not be generalizable to other patient populations or settings. To ensure a safe use of BBM in clinical practice, a process of informed consent, including the provision of adequate information before testing, is appropriate. The clinician should ensure that patients and care partners understand what BBM results can and cannot inform and that the individual desires to be aware of this information. The disclosure of BBM results is likely to have a major impact for some patients and care partners. For this reason, patients and care partners may need emotional support after the disclosure of BBM results. Finally, to be diagnosed with AD remains a source of stigma and discrimination, and this can lead some individuals to delay or forego care. Stigma must be addressed to avoid its effects on patients and care partners and to increase the likelihood that we can realize the potential of BBM.

### Key points about blood-based markers use to aid in diagnosis of AD in clinical practice

1. Trained primary care physicians can use BBM in AD diagnosis without referral to a specialist in memory disorders.
2. BBM such as p-tau measures and Aβ42/40 ratio can be used alone or in combination to aid in AD diagnosis.
3. BBM should be used in combination with clinical and cognitive evaluation. The interpretation of BBM needs to be integrated into the standard process for AD diagnosis (history, cognitive evaluation, structural brain imaging).
4. In primary care, soon (when prospective studies are completed) BBM could be used after a digital cognitive evaluation to assess subjects with objective and persistent cognitive impairment. Prospective studies are needed to validate this use.

## What are the next steps?

Over the past decade substantial progress in AD biomarkers research has been made, and several actions are underway to promote BBM implementation in clinical practice. With few exceptions, almost all current data on BBM performance comes from retrospective studies on batches of samples collected in research settings and in populations with limited ethnocultural diversity and comorbidities. Therefore, it will be critical to demonstrate the effectiveness of BBM when used prospectively and longitudinally with preset cut points in more diverse real-world clinical populations. A close cooperation among pharmaceutical and diagnostic industry partners, academic institutions, regulatory bodies, and patient advocacy associations is needed. It will be useful to continue the Global Biomarker Standardization Consortium, to explore the development of a regulatory focus group with international participation, and to increase efforts to understand medical specialty and primary care needs and constraints.

The advent of BBM with reasonable sensitivity and specificity would lead to substantial changes in health care delivery. We expect to see an important surge in the number of patients seeking potential assessments. There is an urgent need for improved training and proficiency for primary care providers and specialists in memory disorders, as well as a need for development of clinical algorithms to better triage patients from primary care to specialists in memory disorders. Appropriate use criteria in the different settings should be drafted, clearly by defining for each biomarker the clinical purpose, the target population, the potential benefits and risks, and guidance regarding results interpretation and disclosure. There is a crucial need for standardization of operating procedures, to minimize and control pre-analytical, analytical, and post-analytical sources of variability. Work to understand what is needed for reimbursement of BBM is necessary also.

The development of AD BBM is expected to advance into other proteinopathies (TDP-43 pathologies, non-AD tauopathies, alpha-synucleinopathies). This could offer to the clinicians in the future high performance diagnostic tools in the differential diagnosis of cognitive impairment. Further studies with fixed biomarker cut-off set before the study starts need to demonstrate the impact of BBM use on clinical outcomes (e.g., time to diagnosis, diagnosis of AD at an early stage, inclusion in clinical trials, cost/effectiveness, etc).

Large epidemiological prospective studies must be implemented to better understand lifetime development of BBM as well as their interaction with age and comorbidities. Given the high incidence of cognitive decline in people older than 80 years old, more data are needed to interpret such biomarkers in the oldest old. Prospective studies that enroll ethnically and racially diverse individuals are needed. Large prospective studies, in clinical practice must be implemented now to be ready when new therapies are widely accessible. Clear evidence of clinical utility will be required to justify reimbursement.

This is a pivotal moment in the implementation of BBM in clinical trials. Their widespread use should quickly bring dramatic improvements to the conduct of trials, facilitating recruitment, reducing time and costs, and increasing the prospect of effective treatments for the disease. The time needed to implement BBM in clinical routine will inevitably depend on the results of the ongoing clinical trials with DMTs. This process will be accelerated by positivity of one or more clinical trial.

## Figures and Tables

**Table 1.** Some examples of blood-based markers use in clinical trials

Study	Clinicaltrial.gov identifier	Phase	Population	Therapy	Blood biomarker	Role
AHEAD 3-45	NCT04468659	III	Preclinical AD	Lecanemab	Aβ42/40 ratio	Prescreening
AUTONOMY	NCT04619420	II	Early symptomatic AD	JNJ-63733657	p-tau217	Prescreening
BAN2401-G000-201 Core and Open Label Extension	NCT01767311	II	Early symptomatic AD	Lecanemab	Aβ42/40 ratio p-tau 181	Pharmacodynamic
DIAN-TU	NCT04623242	II/III	Preclinical and early symptomatic AD	Gantenerumab	Aβ42/40 ratio p-tau181	Pharmacodynamic
EMERGE	NCT02484547	III	Early symptomatic AD	Aducanumab	p-tau 181	Pharmacodynamic
ENGAGE	NCT02477800	III	Early symptomatic AD	Aducanumab	p-tau 181	Pharmacodynamic
EVOKE	NCT04777396	III	Early symptomatic AD	Semaglutide	p-tau181, NfL, GFAB	Pharmacodynamic
EVOKE-PLUS	NCT04777409	III	Early symptomatic AD	Semaglutide	p-tau181, NfL, GFAB	Pharmacodynamic
INVOKE-2	NCT04592874	II	Early symptomatic AD	AL002	PredvityAD^™^ (algorithm derived from Aβ42/40 ratio, APOE genotype and age)	Prescreening
PROSPECT-ALZ	NCT05063539	II	Early symptomatic AD	LY3372689	p-tau217	Prescreening
TRAILBLAZER-ALZ 2	NCT04437511	III	Early symptomatic AD	Donanemab	p-tau181 p-tau217	Exploratory endpoint
TRAILBLAZER-ALZ 3	NCT05026866	III	Preclinical AD	Donanemab	p-tau217	Exploratory endpoint

Aβ, Amyloid-beta; AD, Alzheimer’s Disease; APOE, Apolipoprotein E; NfL, Neurofilament Light chain; P-tau, phosphorylated tau.

**Table 2.** Potential roles for blood-based markers use in clinical practice

Potential role		Where?
Risk/probability	To detect persons at risk for a disease or condition	Primary care
Diagnosis	To confirm the presence of a disease/condition	Primary care and specialists in memory disorders
Prognosis	To determine risk of worsening and/or progression from MCI to AD	Primary care and specialists in memory disorders
Monitoring	To assess changes in disease severity or the effect of a treatment	Specialists in memory disorders
Pharmacodynamic	To detect changes in response to treatment	Specialists in memory disorders and Clinical trials
Predictive	To predict a favorable or unfavorable effect of a treatment	Specialists in memory disorders and Clinical trials
Safety	To detect an adverse event	Specialists in memory disorders and Clinical trials

**Table 3.** Appropriate and inappropriate uses of blood-based markers in clinical practice

	Appropriate use	Inappropriate use
What blood-based markers?	1. p-tau, Aβ42/40 alone or in combination with other biomarkers, in individuals with typical amnestic presentation 2. NfL to explore neurodegenerative process	1. Any biomarker quantified in an unregulated, non-certified, non-accredited laboratory
When to use blood-based markers?	1. In individuals with objective cognitive impairment (possible or probable AD, MCI/dementia) 2. If suspicion of AD, as part of the initial diagnostic workup 3. If any contraindication or patient aversion to LP (CSF biomarkers)	1. Instead of the cognitive testing 2. In cognitively unimpaired individuals, except context of clinical research 3. Use to determine disease severity in patients having already received a diagnosis of AD 4. APOE4 carriers with no cognitive impairment
Where to use blood-based markers?	1. In primary care to help PCP referring patients to specialists in memory disorders 2. In primary and specialty care to aid in diagnosis of AD (positive biomarkers along with classical cognitive presentation). 3. In clinical trials (Research Setting)	1. In any facility in the absence of trained physicians
How to interpretate blood-based markers?	1. Holistic approach, model combining blood-based biomarkers and cognitive performance 2. Need to perform CSF biomarkers or PET if clinical presentation, structural imaging or other evaluative tests conflict with the blood-based biomarker test result	1. Interpretation of biomarkers without considering the history, clinical exam, cognitive testing, and patient autonomy

Aβ, Amyloid-beta; AD, Alzheimer Disease; CSF, Cerebrospinal Fluid; LP, Lumbar Puncture; PCP; Primary Care Physician; NfL, Neurofilament light Chain; P-tau, phosphorylated tau.
